# Mutation mapping of a variegated EMS tomato reveals an *FtsH*-like protein precursor potentially causing patches of four phenotype classes in the leaves with distinctive internal morphology

**DOI:** 10.1186/s12870-024-04973-1

**Published:** 2024-04-10

**Authors:** Punyavee Dechkrong, Sornsawan Srima, Siriphan Sukkhaeng, Winai Utkhao, Piyanan Thanomchat, Hans de Jong, Pumipat Tongyoo

**Affiliations:** 1 Central Laboratory and Greenhouse Complex, Research and Academic Service Center, Faculty of Agriculture at Kamphaeng Saen, Kasetsart University, Kamphaeng Saen Campus, Kamphaeng Saen, Nakhon Pathom, 73140 Thailand; 2 Center of Excellence On Agricultural Biotechnology: (AG-BIO/MHESRI), Bangkok, 10900 Thailand; 3 Center for Agricultural Biotechnology, Kasetsart University, Kamphaeng Saen Campus, Nakhon Pathom, 73140 Thailand; 4 Scientific Equipment and Research Division, Kasetsart University Research and Development Institute (KURDI), Kasetsart University, Bangkok, 10900 Thailand; 5 Wageningen University, Plant Sciences Group, Laboratory of Genetics, Droevendaalsesteeg 1, 6708 PB Wageningen, the Netherlands

**Keywords:** Leaf variegation, Palisade, Mesophyll, Cell degradation, Chloroplast, *FtsH*, Colour segmentation

## Abstract

**Background:**

Leaf variegation is an intriguing phenomenon observed in many plant species. However, questions remain on its mechanisms causing patterns of different colours. In this study, we describe a tomato plant detected in an M_2_ population of EMS mutagenised seeds, showing variegated leaves with sectors of dark green (DG), medium green (MG), light green (LG) hues, and white (WH). Cells and tissues of these classes, along with wild-type tomato plants, were studied by light, fluorescence, and transmission electron microscopy. We also measured chlorophyll a/b and carotene and quantified the variegation patterns with a machine-learning image analysis tool. We compared the genomes of pooled plants with wild-type-like and mutant phenotypes in a segregating F_2_ population to reveal candidate genes responsible for the variegation.

**Results:**

A genetic test demonstrated a recessive nuclear mutation caused the variegated phenotype. Cross-sections displayed distinct anatomy of four-leaf phenotypes, suggesting a stepwise mesophyll degradation. DG sectors showed large spongy layers, MG presented intercellular spaces in palisade layers, and LG displayed deformed palisade cells. Electron photomicrographs of those mesophyll cells demonstrated a gradual breakdown of the chloroplasts. Chlorophyll a/b and carotene were proportionally reduced in the sectors with reduced green pigments, whereas white sectors have hardly any of these pigments. The colour segmentation system based on machine-learning image analysis was able to convert leaf variegation patterns into binary images for quantitative measurements. The bulk segregant analysis of pooled wild-type-like and variegated progeny enabled the identification of SNP and InDels via bioinformatic analysis. The mutation mapping bioinformatic pipeline revealed a region with three candidate genes in chromosome 4, of which the *FtsH*-like protein precursor (LOC100037730) carries an SNP that we consider the causal variegated phenotype mutation. Phylogenetic analysis shows the candidate is evolutionary closest to the Arabidopsis *VAR*1. The synonymous mutation created by the SNP generated a miRNA binding site, potentially disrupting the photoprotection mechanism and thylakoid development, resulting in leaf variegation.

**Conclusion:**

We described the histology, anatomy, physiology, and image analysis of four classes of cell layers and chloroplast degradation in a tomato plant with a variegated phenotype. The genomics and bioinformatics pipeline revealed a *VAR*1-related *FtsH* mutant, the first of its kind in tomato variegation phenotypes. The miRNA binding site of the mutated SNP opens the way to future studies on its epigenetic mechanism underlying the variegation.

**Supplementary Information:**

The online version contains supplementary material available at 10.1186/s12870-024-04973-1.

## Introduction

Variegated plants display differently coloured or structured areas in leaves and sometimes on stems, flowers, and fruits. Their chimeric or mosaic patterns result from erratic metabolic or developmental processes. Their phenotype may have originated from maternally or nuclear changes or are caused by virus infections, nutrient deficiency, transpositions, or epigenetic phenomena [1]. With stable variegation for consecutive generations, such plants are appreciated for attractive appearance in consumers’ ornamental markets. Plants with variegated foliage are common among herbaceous and climbing species and occur in 1710 angiosperms belonging to 356 genera of 78 families, of which Begonia is the genus with the most cases of variegated phenotypes [2, 3]. In Hara [3], four mechanisms of foliate variegation were distinguished: 1) the pigment-related group that includes the chlorophyll, 2) the pigment type, and 3) the structural-related air-space and 4) the epidermis types. Most studies, however, focus on variegation resulting from chlorophyll deficiency (reviewed in [4]).

For several decades, leaf variegation based on nuclear mutations has been explored at the (ultra)structural, physiological, and molecular levels. Especially the experimental work on variegation mutants in *Arabidopsis thaliana* helped us better understand the underlying mechanisms [1, 5–22]. Other leaf variegation studies were carried out on tomato [23–33], and studies on *Arum italicum* [34], barley [35], *Brassica napus* [36], *Camellia sinensis* [37, 38]; Grapevine [39], *Ilex* X *altaclerensis* [20, 21], *Lotus japonicus* [20], *Epipremnum aureum* [40], tobacco [41], *Hedera helix* [42], *Vigna radiata* [43] and *Zea mays* [44].

One of the common mechanisms involved in variegation is the inactivation of the plastid terminal oxidase of the *immutans* (*im*) found in Arabidopsis. The gene plays a central role in the electron transport chain within [8, 11] and is also essential in chloroplast development and palisade morphogenesis during leaf development. The mutant features a blockage at the phytoene desaturase step of carotenoid biosynthesis. It cannot produce enough coloured carotenoids to avoid photooxidation and is considered a classical carotenoid mutant. The second well-studied variegation is caused by the yellow *variegated*2 (*var*2) gene [45], which encodes the FtsH2 protein that is one isoform of the thylakoid-localised FtsH protease [19]. Further studies suggest that *FtsH* plays a role in the quality control of photosynthetic proteins in thylakoid membranes [41]. Suppression of this gene by RNA interference (RNAi) in tobacco caused variegation in their leaves. Plastid ultrastructure during early leaf development in RNAi-suppressed *FtsH* variegated tobacco revealed that defective plastids accumulate during early leaf development [41] and that thylakoid membranes were dismantled after development. According to [1], the variegation mechanism of *im* and *var*2 in Arabidopsis are essentially different because redundant gene products are proposed to play a role in *var*2 but not in *im*.

The first variegation described in tomato displayed pale green – wild-type green leaf variegation phenotype and was inherited as a dominant maternal trait [23]. A second variegated tomato was characterised by the dominant nuclear gene, located on chromosome 2, which produced variegated leaves with patches of fewer epidermal hairs and abnormal greens [25]. This so-called *Woolly* (*Wo*) mutation is dominant, homozygous lethal and chromosomally unstable. A gene in the woolly mutant induced variegation due to chromosome loss. Lesley et al*.* (1979) [26] hypothesised that the variegated pattern results from a position effect by an inversion involving *Wo*. A comparable case of leaf variegation-associated chromosome instability was studied by Wisman and Ramanna [29], who described an unstable recessive allele of the *yellow virescent* (*yv*^*nw*^) as a nuclear gene affecting chloroplast development that frequently mutates from dominant green to recessive yellow.

Rick et al. [24] described *ghost* (*gh*), an unstable mutant with cotyledons showing partial chlorophyll deficiency and high sensitivity to environmental conditions. In mature plants, leaves are strongly deformed and can be completely devoid of green pigment or display yellow segments containing only 5% chlorophyll. The weak plants that are difficult to retain have been propagated through tissue culture and are used to study pigment biosynthesis and plastid structure during different stages of development [27]. In the *gh* plants, cotyledon and green leaves accumulate carotenoids and chlorophyll, while white leaves only contain phytoene. White tissues of *gh* leaves show an irregular shape of plastids that lack thylakoid membranes and found the impaired thylakoid structure in white fruits, but green tissue exhibits normal chloroplasts with typical internal membranes. *Ghost* mutant plants grown in light showed low mRNA levels of two nuclear genes encoding for chloroplast proteins (*rbcS* and *cab* genes) in white leaves, whereas green leaves are like wild-type. Transcription experiments indicate coloured carotenoids and/or light affect the transcription rates of cytoplasmic mRNAs in green and white leaves of ghost mutants [28]. In a more recent study [31], the *ghost* (*gh*) tomato mutant is orthologous to *im* Arabidopsis, which is abundantly expressed during fruit development in tomatoes. *GH* acts as a chloroplast quinol oxidase and is concerned with chloroplast and chromoplast biogenesis, including pericarp morphogenesis. A *Ds* mutagenesis screen identified an entirely different variegation tomato phenotype [46] and, referred to as the *defective chloroplasts and leaves-mutable* (*dcl-m*) mutation. This unstable mutation, resulting in albino leaves with green sectors, affects chloroplast development and palisade cell morphogenesis. The *dcl-m* mutant phenotype displays light green and dark green sectors in leaf midribs and stems, whereas fruits and petals are wild-type. Leaf palisade cells in albino sectors are not columnar in shape, unlike those in the dark-green sectors, which show a normal shape. The ultrastructure of *dcl-m* leaves indicates that *DCL* protein is essential for chloroplast development, which probably regulates the primary function of the plastid and morphology of palisade cells during leaf development.

A recently discovered mutant was described as a *variegated-leaf* (*vg*) tomato with pale yellow immature leaves and whitening only in the newly mature leaves. The *vg* plants exhibited defective phenotypes in thylakoids and photosynthesis [33]. The *VG* gene in tomato is located on chromosome 7. The defective expression of this gene in ORF10, which encodes thylakoid protein for chloroplast development, impaired chlorophyll synthesis and reduced photosynthesis. It was the only candidate gene whose gene-editing line reproduced the variegated leaf phenotype.

This study presents a new variegation mutant tomato plant found in an M_2_ population of an EMS mutagenised seed sample. Its leaves exhibit segments of dark, medium, and light shades of green, along with white sectors. Stems display green and white paths, whereas cotyledons, flowers and fruits do not show the variegated pattern. We performed brightfield and fluorescence microscopy of leaf cross-sections and transmission electron microscopy of the plastid ultrastructure. We also measured Chlorophyll a/b and carotene content in pools of punches in the four distinguishable leaf segments. To quantify the variegation, we designed a method to digitise such patterns of the four colours in the leaves based on the image analysis Weka colour segmentation machine learning tool. Finally, we use bulked segregant analyses and mutation mapping to elucidate candidate genes that can explain the variegated phenotype. Our studies point to a small genomic region in the long arm of chromosome 4, containing an SNP in the *FtsH*-like gene, a homolog of *Arabidopsis thaliana AtFtsH5* that is involved in photoprotection and thylakoid development [45]. In this study, the allele at the mutation site co-segregated with the variegated phenotype, suggesting it is a candidate gene for the observed variegation patterns.

## Results

### General features of the EMS mutant

**Fig. 1:**
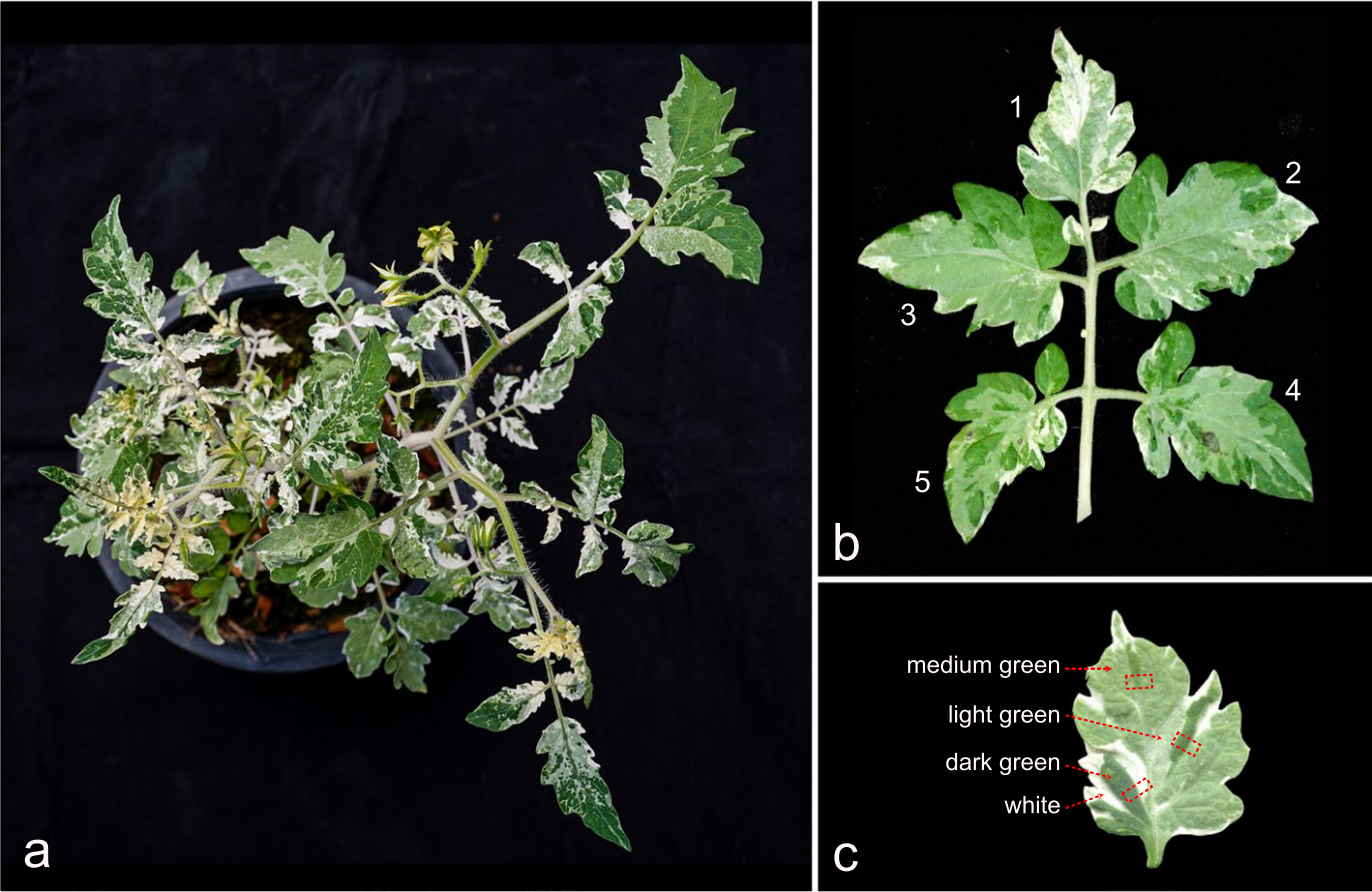
Variegated mutant phenotype tomato. **a** Complete plant; **b** Compound leaf with different variegation patterns in the terminal leaflet (1) and the pairs of lateral leaflets (2,3,4,5). Details of leaves showing Dark Green (DG), Medium Green (MG), Light Green (LG), and White (WH) sectors. **c** A leaflet showing the four different shades of green and white

The plant with a variegated phenotype (M_2_ plant no.433–1), isolated in the M_2_ of an EMS treatment of 2000 seeds, exhibited striking variegated patterns on leaves, rachides, petioles, and sepals (Fig. 1). Leaves displayed arbitrary patterns of dark green (DG), medium green (MG), light green (LG), and white (WH) sectors (Fig. 1c), except for the cotyledon that showed the usual green as that of the wild-type (WT) of the TOMAC463 parent. In addition, sepals, rachides, and petioles incidentally display white-green stripes. (Suppl. Figure S1) We observed low levels of yellowish colouring in young, developing branches (Fig. 1a). Differences in the variegation patterns between the terminal, lateral primary, and secondary leaflets are apparent (Fig. 1b), but this differentiation shape does not hold for other leaves. A quantification method to describe these differences is detailed below.

Reciprocal test crosses with the wildtype TOMAC463 and the M_2_ variegated plant No. 443–1 revealed uniform WT progeny (20 plants for each cross), whereas selfings of the mutant plants gave completely variegated offspring. Crossings of the heterozygote in both reciprocal crosses produced wildtype and variegated offspring segregating in a 3:1 Mendelian ratio (Table 1), suggesting that a nuclear recessive mutation caused the phenotype.

**Table 1:** Genetic Analysis of the reciprocal crosses between the TOMAC463 wild-type and M_2_ No.443–1 tomato-variegated parents

Cross	No. of wildtype plants	No. of variegated plants	Genetic Ratio	Χ^2^
TOMAC463 x M_2_ Plant No. 443–1, F_2_	160 (152.25)	43 (50.75)	3:1	1.58
M_2_ Plant No. 443–1 × TOMAC463, F_2_	108 (103.5)	30 (34.5)	3:1	0.78

Significance χ^2^ = 3.84 at P_*df*=*1*_ = 0.05

### Histology in brightfield and fluorescence micrographs

**Fig. 2:**
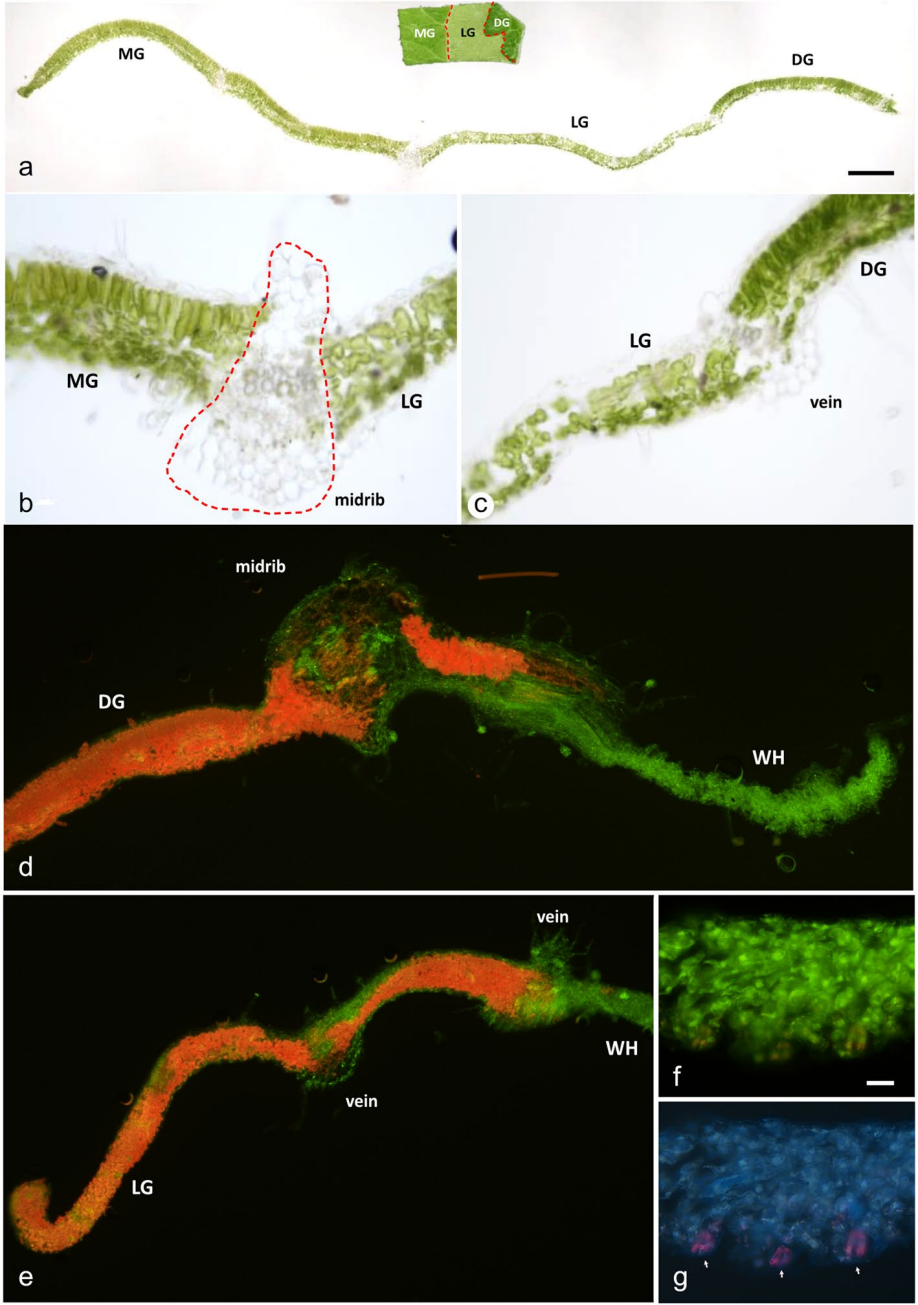
Cross-section of the variegated tomato leaf. **a** Overview stitched image of MG-LG-DG cross section. **b** (Left) the high magnification image of the cross-section of MG and LG sectors in brightfield microscopy, the red-dash line presented the midrib of the variegated leaf. **c** (Right) Higher magnification of the cross-section between LG and DG sector of the variegated leaf in brightfield image. **d** The fluorescent image showed the cross-section between the DG and WH sectors. **e** The cross-section image of the variegated leaf showed the transitional zone from LG to WH sectors. **f** The fluorescent image taken with a broadband filter for blue/green (U-MWU2) of the WH sector at high magnification is not clearly visible in the stomata with an orange colour. **g** The fluorescent image of the WH sector, which is taken with a broadband filter for green/red (U-MWB2), exhibited the stomata in a pink colour (arrows pointed at stomata)

We first studied the topological patterns of cell layers in transverse sections of the variegated leaves, with particular attention to leaf punches that contain two or more different leaf classes (Fig. 2a, inset). Our first series followed a straightforward protocol based on glutaraldehyde fixation, agarose embedding, and sectioning with a vibrating blade microtome to make 30 µm transverse sections for bright field microscopy. We captured images at different focal planes and combined them by stacking. Other fixations based on formaldehyde and formaldehyde/glutaraldehyde fixations [47, 48] were also tested but gave dull and weak fluorescence signals (data not shown). Figure 2a shows an overview of a leaf cross-section obtained by stitching three adjacent low-magnification bright field images. The border between medium and light coincides with a sizeable colourless midrib (Fig. 2b), whereas in a small vein between the light and dark green, a much smaller colourless transition is perceived (Fig. 2c). We obtained characteristic autofluorescence and glutaraldehyde-induced fluorescence in the cellular layers of the leaves, demonstrating internal anatomic structures that are helpful in the characterisation of the four colour classes (Fig. 2d, e).

In the WT leaves, fluorescence images of the cross-section displayed brightly orange tissues in our broadband filter, in which palisade mesophyll cells are columnar shaped and spongy cells irregular (Suppl. Figure S2a). Vascular bundles are less orange, whereas epidermal cells lack entirely chloroplasts. In the DG sectors, leaves are slightly more compact than those of WT (Table 2) and are often bulged. Palisade and spongy mesophyll cells of the DG sectors are distinguishable and like WT (Fig. 2d). However, the spongy layers of DG sectors are larger than those of WT, but this difference is not conspicuous. The intracellular space of the DG spongy layers is more prominent than in the WT. The mesophyll layer of MG sectors also features elongated palisade cells with well-arranged and dispersed spongy cells still well-defined beneath the lower layer of palisade cells (Suppl. Figure 2c). We often observed larger intracellular space between the palisade cells. The borders between MG and LG sectors are often delineated by smaller veins or at midribs (Fig. 2b and Suppl. Figure 2b). Still, their transition between the different classes can be gradual (Suppl. Figure 2d). Palisade and spongy cells are identified in the LG sectors based on their position in the leaf but have comparable numbers of chloroplasts. Palisade cells are deformed and now resemble the shape of spongy cells (Fig. 2b, c, e). The size of intracellular space in the mesophyll layers is often larger than in the DG sectors. The thickness of the WH sectors is quite variable; tissues are degraded, and cells are entirely devoid of chloroplasts (Fig. 2d). Palisade and spongy cells have lost their identity, whereas midribs and veins produce bright autofluorescence (Fig. 2d, e). Chloroplasts are completely lacking except in the stomata cells (Fig. 2f, g). In addition, the leaf layers’ architecture in the transition zone in Fig. 2d displays a border between a DG and a WH segment at a midrib of the leaf. Parenchyma tissues in the midrib contain an orange hue of a few chloroplasts in and around vascular tissue, including veins, but are absent in the xylem and phloem vessels. The polarity of the palisade and spongy cells no longer exists, and cells are disrupted. In contrast, only a short part of the mesophyll tissues with decreasing numbers of chloroplasts continued into the WH zone. In Fig. 2e, a comparable disruption at the border of LG and WH was observed. Such gradual transition zones, often measuring 30 – 100 μm, were observed regularly between the borders of all classes but were most noticeable at sectors neighbouring the WH sectors. Other examples of aberrant transition zones are shown in Supplementary Figure S1, where the gradual transitional between MG and LG segments is displayed in Suppl. Figure S1c. In contrast, Supplementary Figure S1d shows that the WH segment has grown beneath the palisade cell layer of the green sector. This green sector only contains palisade cells.

### Anatomy of the five classes in semi-thin sections

**Fig. 3:**
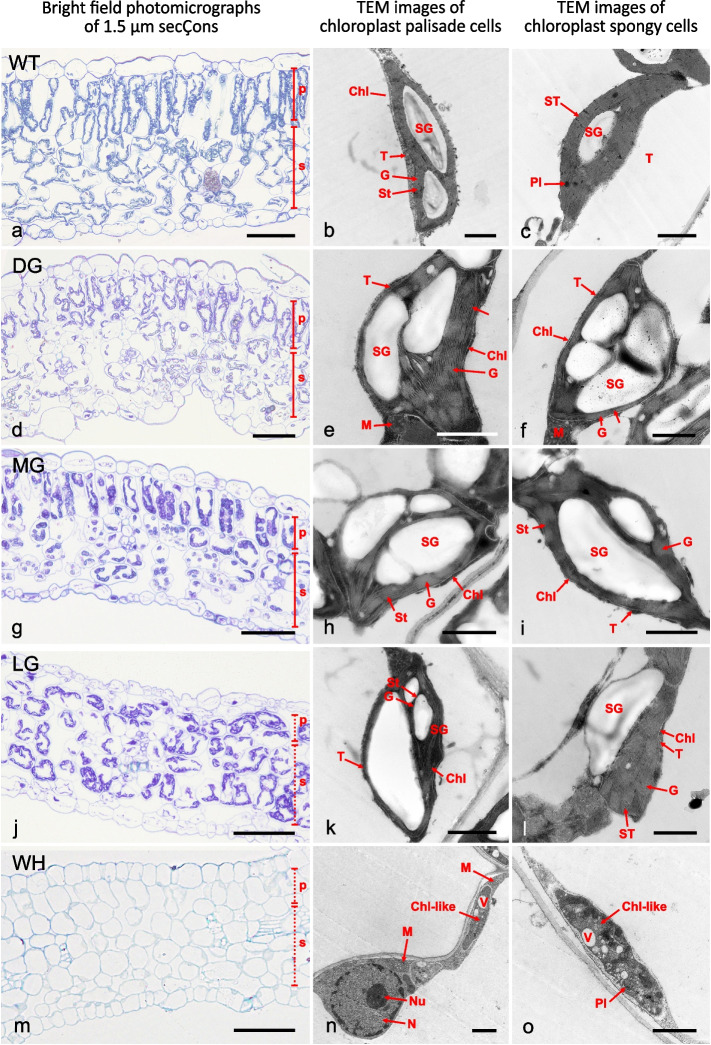
Histology LM of the wild-type and the four colour types in the variegated leaves (WT-DG-MG-LG-WH). **a**, **d**, **g**, **j**, and **m**. Light microscopy toluidine blue staining of the WT, DG, MG, LG, and WH sectors semi-thin section. **b**, **e**, **h**, **k**, and **n**. Ultrastructure of plastids in palisade cells of WT, DG, MG, LG, and WH. **c**, **f**, **i**, **l**, and **o**. Plastid ultrastructure of WT, DG, MG, LG, and WH sectors in spongy cells. Chl, chloroplast; Chl-like, chloroplast-like structure; G, granum;; M, mitochondria; N, nucleus; Nu, nucleolus; P, palisade cell layer; Pl, plastoglobuli; S, spongy cell layer; SG, starch granules; ST, stroma thylakoid; T, tonoplast; V, vacuole; scale bar in the **a**, **d**, **g**, **j**, and **m** = 50 µm; scale bar in the **b**-**c**, **e**–**f**, **i**, **k**-**l**, **n**–**o** = 1 µm

Next, we analysed the tissue anatomy and cell structure in 1–1.5 μm embedded cross sections in the WT and four classes of the variegated sections. Representative examples for each class are shown in Fig. 3a, d, g, j, and m, and an overview of the measurements is presented in Tables 2 and 3. Leaves and spongy mesophyll layers are thicker in the DG sectors, while the highest value of the palisade cell layer is in the WT segments. In addition, the ratio of palisade cells/spongy cells is more significant in LG (Table 2). No striking differences were observed in the number of palisade cells of all segments, including the WT. In contrast to palisade mesophyll cells, the highest number of spongy cells was significantly shown in the WH sectors (Table 3). To assess the difference in the anatomical analysis of the five classes, we compared the average value of each segment as a percentage. In DG sectors, the average leaf thickness and spongy cells are about 5% and 4.5% thicker than WTs, whereas palisade cells of WT are larger than DG cells by about 22% (Suppl. Table ST1). Furthermore, palisade cells of DG sectors were less elongated, often almost spherical, in which both palisade and spongy cells contained more chloroplasts and even seemed more than in WT leaves. The intercellular space between palisade cells is much less, but between the spongy cells, the space is comparable to the WT (Fig. 3d). The mesophyll of MG sectors showed elongated palisade cells and well-arranged and had more intercellular space among them (Fig. 3g). The average palisade cell size of MG is 37.09 µm, larger than that of WH, except for the others. Its spongy cell thickness is larger than that of LG. The ratio of palisade cells/spongy cells is not different from that of WT and DG (Table 2). The number of spongy cells does not differ from the others, except for the WH sector (Table 3). In LG sectors, palisade and spongy cells are hardly distinguishable (Fig. 3j). Those mesophyll cells are about the same size and have less space in the intercellular, and vascular cells are highly condensed. In contrast, the palisade and spongy cell ratio is approximately 32% higher than the others (Suppl. Table ST1). The number of cells is also not different from the others, except for WH (Table 3). The leaf thickness of the WH sectors is also nearby that of LG and MG. The palisade cell layer is the lowest, while its spongy layer is close to the WT and DG sectors (Table 2). The number of spongy cells is also the highest (Table 3). Intercellular space can be observed in the mesophyll cells, in which both palisade and spongy cells almost show empty and do not contain any chloroplasts (Fig. 3m).

**Table 2:** Histological characteristics of the WT and the four shades of green and white (DG, MG, LG, and WH) in the variegated tomato leaves

Type	Leaf Thickness (µm)	Thickness of Palisade Cell layer (µm)	Thickness of Spongy Cell layer (µm)	Palisade Cells/Spongy Cells (µm)
WT	158.16 ± 9.01^a^	54.94 ± 3.83^a^	76.46 ± 9.12^a^	0.74 ± 0.10^b^
DG	167.13 ± 14.39^a^	44.89 ± 9.30^b^	80.09 ± 22.18^a^	0.57 ± 0.10^c^
MG	118.16 ± 4.98^b^	37.09 ± 2.09^c^	59.56 ± 4.79^b^	0.63 ± 0.07^bc^
LG	123.12 ± 3.50^b^	48.55 ± 7.65^ab^	45.74 ± 5.27^b^	1.09 ± 0.12^a^
WH	121.82 ± 2.53^b^	26.81 ± 4.55^d^	74.14 ± 7.28^ab^	0.39 ± 0.12^d^

Letters for each parameter point at significant differences at *p* ≤ *0.05*. WT refers to the wildtype TOMAC463 variety, and DG, MG, LG, and WH to dark green, medium green, light green, and white leaf sectors, respectively

**Table 3:** The number of palisade and spongy cells in the mesophyll layer of the different parts of the variegated tomato leaves

Type	No. of palisade cells	No. of spongy cells
WT	33.80 ± 3.03	50.60 ± 6.73^b^
DG	49.14 ± 16.41	72.00 ± 11.68^ab^
MG	33.83 ± 12.62	69.00 ± 23.20^b^
LG	49.00 ± 5.48	68.75 ± 12.55^b^
WH	36.50 ± 8.74	93.75 ± 23.46^a^

The counted number of palisade cells in all samples is tested statistically by Duncan's Multiple Range test (DMRT). Means values followed by different letters for each parameter are significantly different at *p* ≤ *0.05*. DG refers to the dark-green area of the leaf, MG to the medium-green area of the leaf, LG to the light-green area of the leaf, and WH to the white area of the leaf. Meanwhile, WT represents the wild-type of TOMAC463 phenotype

### Ultrastructure chloroplasts

Transmission electron photomicrographs of the chloroplasts in the palisade and mesophyll cells of the WT, DG, MG, LG, and white sectors are displayed in Fig. 3, second and third columns, respectively. Chloroplasts in mesophyll cells of WT, DG, MG, and LG sectors are chloro-amyloplast types, as the size and shape of the mature chloroplasts are proportional to the size of starch granules inside the chloroplast. Chloroplasts of WT and DG, MG, and LG sectors show abundant thylakoid membranes and dense grana stacking. In addition, WH chloroplasts in mesophyll cells are lacking (Fig. 3n, o). Plastoglobuli were observed in the WT and the WH chloroplasts (Fig. 3c, o). Larger starch granules were found in the DG and MG cells, in the chloroplasts of palisade and spongy cells (Fig. 3e, f, h, i), while part of the thylakoids varies depending on the starch granule deposition. In the WH sectors, abnormal plastids are found in the mesophyll cells. They are flat structures attached to the cell wall, completely lacking thylakoid membranes and grana structures (Fig. 3n, o). Large and small vacuoles, as often found in the chloroplast-like structure, are shown in Fig. 3o.

### Chlorophyll measurements

**Fig. 4:**
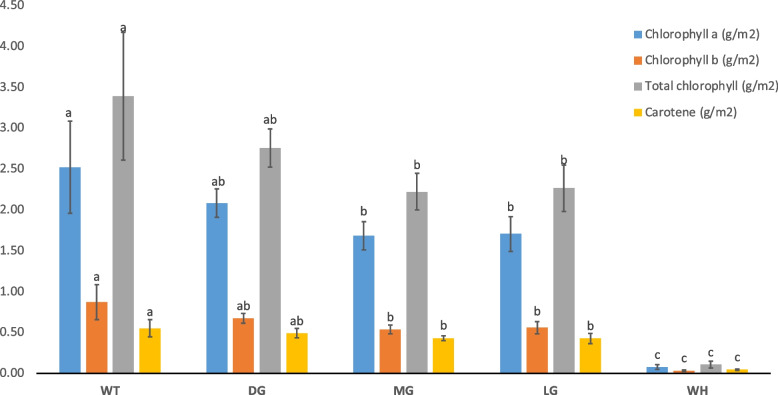
Spectrophotometry of chlorophyll and carotene content in which the x-axis indicates the concentration of chlorophyll and carotene (g/m^2^) and the y-axis displays the four sectors of the variegated leaves, including the wild-type plants. The same letters in each bar mean no significant differences at *p* < 0.05 and * = *p* < *0.05* by DMRT. Error bars represent the standard deviation of mean value from triplicates for each biological sample

The amounts of chlorophyll *a*/*b* and carotenoids follow the decrease in colour intensity of leaf segments proportionally. Leaf punches collected from the DG, MG, LG, and WH tissues had lower total chlorophyll content of 81.3, 65.5, 67.7, and 3.2% compared to the WT (Fig. 4). Chlorophyll *a* and *b* contents were not significant among the variegated green segments (DG, MG, and LG), but MG and LG differed significantly from the WT, and the same holds for the carotenoid. The measurements also revealed that the pigments in the WH sectors were very low and differed significantly from the other classes and WT.

### Quantification of variegation patterns

**Fig. 5:**
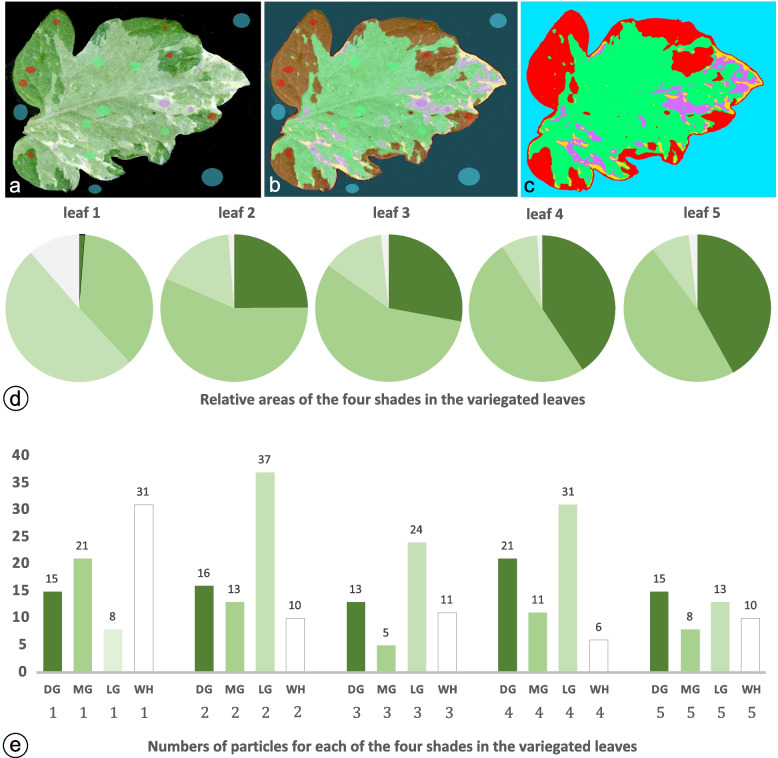
Weka colour segmentation of leaflet 2 (see Fig. 1b). **a** shows an example of small selections for each class (DG, MG, LG, and WH), along with the black background. **b** shows the overlay with the segmented colours. **c** displays the produced classified image with indexed colours for each class. This image, converted into an RGB image, was used for colour thresholding and particle analysis. The resulting pie chart in **d** shows a graphical representation of areas for the green and white segments of the five leaflets, whereas **e** shows column charts with the numbers of coloured segments in each leaflet

We could always distinguish three shades of green and white in the variegated mutant's leaves. These classes were easily discernible upon visual inspection, but a closer look made clear that subtle differences in hue could be observed in each. We realised that describing the coloured patterns in the leaves would generate complex data sets unsuitable for comparative studies on the effects of plant position, developmental processes, and environmental influence. To reduce the colour complexity, we developed a system of colour segmentation in which the computer learns to define ranges for each class of green and white that combines leaf sectors of more or less the same shade. The Weka colour segmentation system, initially designed for coloured microscopic structures [49], enables various coloured patches into pseudo-coloured images with indexed colours for DG, MG, LG, WH, and Background classes. As an example, we used the compound leaf in Fig. 1b, showing a terminal leaflet (1) and the pairs of lateral leaflets (2–5) [50]. The small folioles between leaflets along the rachis or on either side of the petioles were not considered. Each leaflet was now imported onto the Weka colour segmentation plugin, and small pixel areas for each class were defined to train the system until the pseudocolours overlay corresponded entirely with the variegation patterns (Fig. 5a and b).

The classifiers thus obtained from leaflet two were used for the other leaflets and produced five indexed images of the variegated leaflet patterns (Fig. 5c). For each leaflet, we converted the indexed colour image into RGB colours and selected each colour-by-colour thresholding, followed by particle analyses. Two variables were considered: 1. Measuring the total area of the pixels for each colour and 2. Counting the number of sectors for each colour, with the restriction that minor sectors, e.g., with less than 100 pixels, were omitted (Suppl. Table ST2). In Fig. 5d, we showed pie charts of the relative areas of the DG, MG, LG and WH classes, whereas in Fig. 5e, the absolute numbers of each class were shown for the five leaflets. Striking observations include the high number of white sectors in Leaflet 1 (31) and the low number in Leaflet 4 (6). In contrast, in Leaflets 4 and 2, the numbers of DG sectors dominate (21 and 16, respectively). As the total area of the sectors, leaflet 4 has the highest area of DG and the lowest in Leaflets 1 and 5. The class of MG is highest in Leaflet 1 (21), while that of LG is highest in Leaflet 2 (37). In this way, numerical representations of the variegation patterns, as seen in Fig. 1b, are now accurately demonstrated, enabling us to compare with more significant numbers of leaves grown under different development and environmental conditions.

### Bulk segregant analysis, genomics, and mutant mapping

**Fig. 6:**
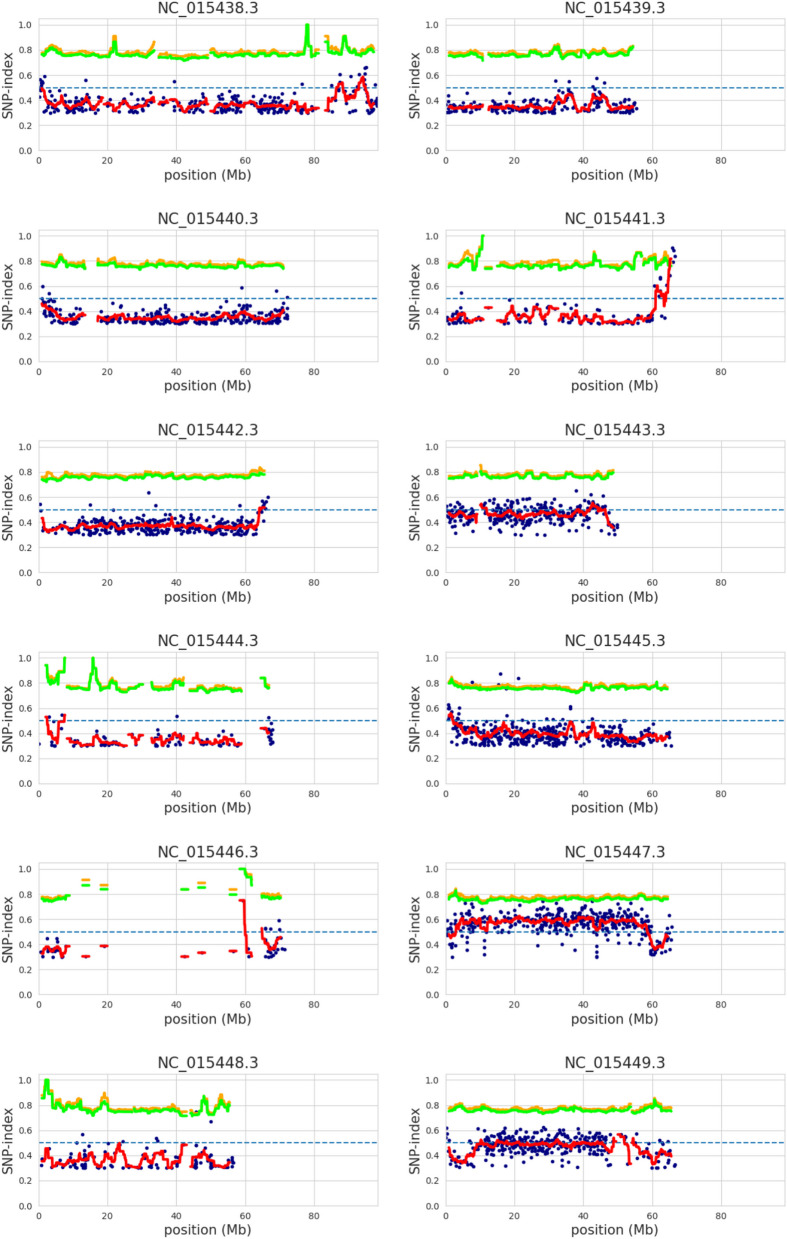
MutMap chromosome plot representation of the distribution of SNPs across the chromosomes. BLUE dot: variant, RED line: mean SNP-index, a critical visualisation indicated significantly enriched in mutant bulk compared to wildtype, which identifying genomic regions on Chromosome 4 (NC_015441.3) as associated region with the variegated phenotype., ORANGE line: mean p99 (99% confidence interval of simulated SNP-index) SNPs that have a mutant allele frequency above the p99 threshold are considered significantly enriched in the variegated population then the candidate regions associated with variegated were identified in this region., GREEN line: mean p95 (95% confidence interval of simulated SNP-index), similar to p99 but consider confidence at 95%

Whole-genome sequencing of the wildtype phenotype and variegated parent, F_2_ wildtype-like bulk, and F_2_ variegated bulk was used to explore a casual mutation linked to the variegated phenotype. The bulk population was collected from 50 plants of F_2_ from the reciprocal population for each type. The parent individuals and bulks were sequences using standard pair-end short-read nanoball technology. The sequences were aligned to a tomato reference genome (SL3.0, https://solgenomics.net/organism/Solanum_lycopersicum/genome). The variation linked to variegated was identified using MutMap, a mutation mapping algorithm developed by Abe and colleagues [51]. An SNP index was calculated as the ratio between the number of reads of mutant SNP and the total number of reads corresponding to the SNP in a sliding window approach (moving averages). The chromosome-wide mapping is shown in Fig. 6. A variation with SNP-index 1 or close to 1 indicates variation linked to causal mutation. We found 335 genes in the linked sliding window region covering 2.4 Mb region, ranging from 64,100,000 to 66,500,000 bp detected by MutMap (Table 4). Only four InDel and three SNPs were detected in the linked windows region located in three genes with a 99% confidence interval of simulated SNP-index in a region of sliding window analysis peaked.

**Table 4:** Sliding_window with 99% confidence interval as detected by MutMap. The position resulting from the sliding window analysis indicates the centre position of the window size at 2 Mb

CHROM	POSI	mean_p99	mean_p95	mean_SNPindex
NC_015441.3	65100000	0.7852	0.7572	0.7944
NC_015441.3	65200000	0.7846	0.7599	0.8168
NC_015441.3	65300000	0.7846	0.7599	0.8168
NC_015441.3	65400000	0.7846	0.7599	0.8168
NC_015441.3	65500000	0.7846	0.7599	0.8168

**Fig. 7:**
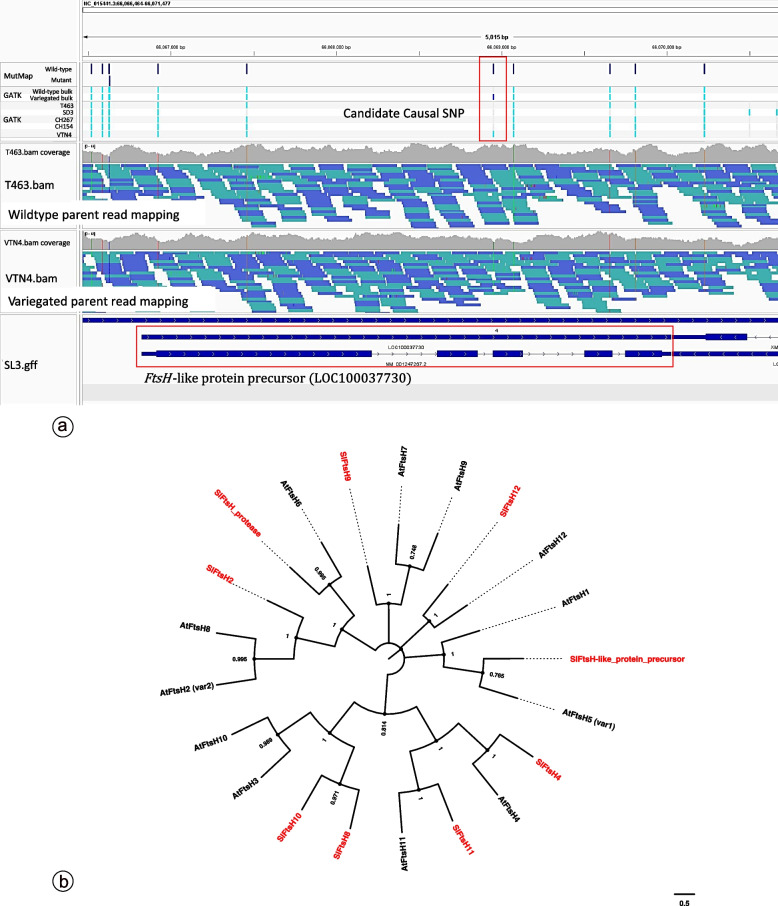
**a** displays candidate causal SNP in the *FstH*-like gene. The red box in the upper track shows SNP detected by MutMap (wild-type bulk and variegated bulk) and SNP detected by individual wildtype and variegated plants. The read mapping coverage for wild-type and variegated parents is displayed from the bam file in the middle tracks. The structure of the *FstH*-like gene was illustrated in the bottom track. **b** illustrates the phylogenetic study was inferred using the Maximum Likelihood method with 1,000 bootstraps. The percentage of trees in which the associated taxa clustered is shown next to the branches

We identified the following candidate genes out of 335 genes located in the linked sliding window region as shown in Table 5: PREDICTED: *Solanum lycopersicum* peroxidase 9 (LOC101264212), *Solanum lycopersicum FtsH*-like protein precursor (LOC100037730) and PREDICTED: *Solanum lycopersicum* BTB/POZ domain-containing protein At2g30600 (LOC101258455). However, only the SNP in the *FtsH*-like protein precursor was identified by variant calling protocol using the GATK best practice pipeline [52]. Figure 7a illustrates the candidate SNP position highlighted in the red box. The upper track shows alleles identified by MutMap. The second track displayed variants identified by GATK of both bulk (wild-type vs. Variegated) and individual plants in different phenotypes; T463 (TOMAC463), CH154, CH267, and KUPINK649 are wild-type VNT4 are variegated plants. The middle tracks T463(TOMAC463).bam and VTN4.bam illustrate read mapping for wild-type and variegated bulk, respectively. The bottom track illustrated *FtsH*-like protein precursor (LOC100037730) annotation. The evolution analysis by the Maximum Likelihood method using the candidate FtsH-like protein precursor (LOC100037730) protein along with eight other tomato *FtsH* and twelve Arabidopsis *FtsH* shows that the candidate is evolutionary closest to *VAR*1 (Fig. 7b). The bootstrap consensus tree inferred from 1000 replicates represents the evolutionary history of the taxa analysed. The evolutionary distances were computed using the Poisson correction method and are in the units of the number of amino acid substitutions per site. Initial tree(s) for the heuristic search were obtained automatically by applying Neighbor-Join and BioNJ algorithms to a matrix of estimated pairwise distances using the JTT model and then selecting the topology with a superior log likelihood value. This analysis involved 13 amino acid sequences. All ambiguous positions were removed for each sequence pair (pairwise deletion option). There were a total of 1059 positions in the final dataset. The analysis indicated that this candidate *FtsH*-like protein precursor (LOC100037730) is evolutionary closest to *VAR1*.

**Table 5:** SNP and indel with a 99% confidence interval of simulated SNP-index that were identified in sliding window regions

CHROM	POSI	variant	depth	p99	p95	SNP index	Blast hit
NC_015441.3	64710996	indel	25	0.84	0.84	0.96	-
NC_015441.3	64793524	indel	52	0.7885	0.7885	0.9615	-
NC_015441.3	64944775	indel	26	0.8462	0.8462	0.8462	PREDICTED: Solanum lycopersicum peroxidase 9 (LOC101264212), mRNA
NC_015441.3	65160699	indel	60	0.7833	0.7667	0.85	-
NC_015441.3	65732119	snp	73	0.7808	0.7534	0.9041	-
NC_015441.3	66068953	snp	68	0.7941	0.7647	0.7941	Solanum lycopersicum *FtsH*-like protein precursor (LOC100037730), mRNA
NC_015441.3	66170258	snp	69	0.7826	0.7681	0.8841	PREDICTED: Solanum lycopersicum BTB/POZ domain-containing protein At2g30600 (LOC101258455)

The sequencing analysis has discerned the presence of the alternative allele at candidate SNP positions, where all variegated plants exhibit homozygosity for the A allele, contrasting with the G allele observed in normal leaf plants. Furthermore, a notable heterogeneity in the F_2_ population of normal leaf plants is observed with both A and G alleles at this specific position, as depicted in Suppl. Figure S3.

## Discussion

This study described the genetics, anatomy, ultrastructure, and genomics of a plant with a variegated phenotype found in the M_2_ of an EMS mutagenised tomato seed sample. The variegation in the leaves caused by a recessive nuclear mutation displays irregular patterns of dark, medium, and light green and entirely white sectors, except the cotyledons, which are WT green. The DG segments clearly differ from the WT green, making five classes to compare their histological, physiological, and microscopical features. The Mutmap analysis based on genomic comparisons of pooled DNA from plants with mutant and wildtype phenotypes [51, 53, 54] revealed that an *FtsH-like* protein precursor located on chromosome 4 is the most likely candidate. Taking these results into account, we concluded that this *FtsH* variegation mutant is dissimilar from the previously published tomato mutants with variegated leaves, including studies of a maternally inherited variegated tomato [23]; the *Woolly* mutant [25]; a recessive allele of *yv* (*yv*^*mut*^) [29], a cytoplasmic or mitochondrial mutation [55]. More recent studies on chromosomal mutations in the tomato genome are the *Ghost* phenotype [24, 27, 28, 31], *DLC* [46], and *VG* [33]. None of them resembles the variegation described in our study. However, in *Arabidopsis thaliana*, the variegation mutants with altered *FtsH* control are the *var*1 and *var*2 [1, 12–14, 19, 41, 56–60], showing that our variegation mutant is an orthologue of these genes. The phylogenetic analysis indeed confirmed that this tomato mutant is closest to *VAR*1.

The content of photosynthetic pigments in different colour segments of variegated leaves revealed that visible change in phenotype is caused by reduced or absent pigment due to chlorophyll deficiency. Likewise, for many ornamental plants, the variegated features are caused by a chlorophyll deficiency in one or two of the three cell layers or a disruption in chloroplast development [21]. Plant leaf colour changes are caused by genetic mutations that directly or indirectly affect chlorophyll biosynthesis and degradation and can alter the chlorophyll content [61, 62]. These reasons can explain why the WH segment contained very low pigments and undeveloped chloroplast. The ratio of Carotene and Chlorophyll a + b in WH sectors was higher than the other classes and WT, indicating a more robust photoprotective function (in guard cells or vacuolated plastids). This result correlated well with the study of [63], who showed that the white sector of variegated leaves had a higher ratio of Carotene and Chlorophyll a + b. Zhao et al. (2020) [36] reported that the disruption of carotene biosynthesis also leads to abnormal plastids containing the globular vacuolated membrane structure in the white sectors of leaves. However, there was a decreasing trend in Chlorophyll a compared to Chlorophyll b, as seen in the DG sector. This may compensate and minimise the loss of Chlorophyll a, the primary pigment in photosynthesis.

In addition, the ratio of Chlorophyll a/b generally indicates a positive correlation with the PSII core ratio to the light-harvesting of chlorophyll–protein complex [64, 65], which this ratio of three green sectors is more than 3. As per our data, we can suggest that the green sectors of our mutant may compensate for the photosynthesis in WH sectors and enhance the photosynthesis capability. This corresponds to the results of [9], which reported that the higher ratio of Chlorophyll a/b in green sectors of *im* mutant enhanced the photosynthetic potential, compensating for the lack of photosynthesis of WH sectors. This adaptation phenomenon is to avoid light stress. Surprisingly, we noticed abnormal mesophyll cells in the LG segments (Figs. 2c and 3j). Palisade cells with low pigments are deformed and do not enlarge to their columnar shape, resulting in a loose connection between palisade cells and more air spaces. These characteristics are related to the structural variegation mechanism explained in some variegated plants, where the pale green area is caused by the diffuse reflection of light from air spaces under the epidermis [2, 66, 67].

The morphometric features of the four shades are summarised in Tables 2 and 3. Leaf thickness in the DG sectors is 5.7% higher than the WT and can be explained by a 4.7% thicker layer of spongy cells. However, cell density in the palisade and spongy cell layers is much higher, with 45% and 42% more cells, respectively. The leaf thickness of the MG, LG, and WH sectors is now reduced by more than 22%. The thickness of the palisade in the LG sector is about the same as WT and shows a palisade/spongy ratio of more than 1. The number of palisade cells is highest in DG and LG, whereas spongy cells are highest in DG and WH sectors. TEM images of the chloroplasts showed little or no dramatic changes in the three green-shaded sectors. Starch granules were abundant in DG, MG, and LG, although chloroplasts in the palisade and spongy cells of both DG and MG were larger than those observed in the WT and LG sectors. The chloroplast ultrastructure in DG, MG, and LG segments was indistinguishable. Thylakoid membranes were abundant, and grana stacking was dense in the DG, MG, and LG chloroplasts, as in WT. Unlike the WH sector, chloroplasts are abnormal in the mesophyll cells, completely lacking thylakoids and grana structures, and sometimes with big vacuoles. Abnormal plastids, which displayed chloroplast-like structures, were formed along with leaf development. As a result, it suggests that plastids in our WH segment are impaired in their ability to form thylakoid membranes and are irreversible phenomena once developed in which chloroplast biogenesis is inhibited during leaf development. In principle, the main stage of the plastid differentiation stage is thylakoid formation which, if it fails to form a thylakoid membrane, leads to failure of the chloroplast conformation [13, 14, 33, 68]. Plastids in leaf cells that fail to produce thylakoid structure during leaf development typically result in variegated patterns [13, 68, 69].

### Quantifications of variegation pattern by Weka colour segmentation

Variegation patterns of four shades between the leaflets of the same compound tomato leaf [50] are arbitrary and very dissimilar and differ between different parts of each plant, between plants and under different environmental conditions. In [70], computerised extraction of morphological and geometrical features was developed for plants with compound leaves, but this method has yet to be designed mathematically to describe variegation phenotypes. Therefore, we applied the Weka colour segmentation widely used in microscopic analyses [49] for digitisation and measuring pixel areas of the four shades in the leaflets. As proof of principle, we demonstrated differences in relative areas and numbers of sectors as primary parameters.

The relative proportion of these shades in the five leaflets, as shown in Fig. 4, may suggest some preference for one of the classes given certain unknown developmental or environmental conditions. Still, other leaves demonstrate different patterns (data not shown), meaning a general conclusion could not be drawn. The second parameter reveals a measure of the rate at which primordial cells switch from one status to the other. This switching variable seems arbitrary, and general statements on controlling this switching parameter are still debatable and need further research. In addition, color segmentation and particle analysis extend morphometric variables such as sector symmetry, boundary length, and smoothness to future large-scale quantitative measurements of variegation patterns under well-defined environmental and developmental conditions incited by abiotic and biotic stresses. Our method is one step further to versatile tools than the variegation percentage quantification by [71] based on Photoshop filtering and pixel calculations. A different albeit exciting approach is that of [72], monitoring changes in RGB colours and variegation patterns.

### Implications of the identified Ftsh-like precursor gene

To classify the potential role of the candidate *Ftsh*-like precursor gene identified by MutMap, we used twelve *Arabidopsis FtsH* and nine *Solanum FtsH* genes for phylogenetic analysis, which was inferred using the Maximum likelihood method. Both *VAR*1 and *VAR*2 have been proposed to have significant involvement in breaking down photodamaged subunits within photosystem II. They encode similar FtsH metalloproteases (*FtsH5* and *FtsH2*, respectively), which form oligomeric complexes in the thylakoid membrane for normal chloroplast function and green sector formation ([1, 58, 73, 74]). According to the phylogenetic analysis result, the *SlFtsh*-like precursor gene might be an ortholog of *VAR1,* which potentially has a function in the disruption of the thylakoid development process. However, the SNP identified by MutMap in this gene was annotated as a synonymous SNP. Other possible molecular biological controls of gene function, such as epigenetic regulation or transposon, exist. A recent study [75] reported that PTC-miR6478 targeted the ATP-dependent zinc metalloprotease FtsH mRNA. In this study, the alternative allele identified through MutMap in candidate *FtsH*-like protein precursor (LOC100037730), as shown in Suppl. Figure S3 potentially changed the possible miRNA binding site, which is predicted by miRDB and TAPIR. It hypothetically disrupts the photoprotection mechanism and thylakoid development, resulting in leaf variegation. The scientific community is paying substantial attention to these miRNAs due to their recognised role in gene expression control, as discussed by Bajczyk M. et al*.* (2023) [75]. Therefore, we proposed the homozygous A allele in *Solanum lycopersicum FtsH*-like protein precursor (LOC100037730) identified in this study as the candidate allele of variegated traits in tomatoes, that potentially be the target of miRNA6478 which deserves consideration for future studies.

## Methods

### Mutagenesis population

A total of 2000 seeds of TOMAC463 tomato, harbouring a tomato mosaic virus resistant gene (*Tm-2a*), was treated with 1% Ethyl MethaneSulfonate (EMS) to generate a mutant population. M_1_ seeds were reared in the Tropical Vegetable Research Center (TVRC, at the Kamphaeng Saen Campus), and the M_2_ seeds from 30 fruits per plant. We selected M_2_ seeds of 325 accessions (ten seeds/accessions) to evaluate their phenotypes in an evaporative cooling greenhouse at TVRC. One of these plants (No. 433–1) displayed the variegated leaf phenotype. We crossed the wildtype tomato (TOMAc463) with variegated (No. 433–1) and reciprocal parents for the genetic analysis of the trait. F_1_ and F_2_ generations of each crossing were grown in a net house at TVRC, Kasetsart University, Kamphaeng Saen Campus for evaluating phenotypes and harvesting seeds.

We cut pieces of about 1 × 1 cm from the variegated leaves, containing sectors of different green shades and white tissue. These cuttings were fixed in 2% glutaraldehyde in 0.1M sodium cacodylate buffer, pH 7.4, and embedded in a 3.5% agarose gel at temperature and duration. With the Leica VT1000S Vibrating Blade Microtome, we made 30 µm transverse sections for bright field microscopy (Olympus BX51 fluorescence microscope).

### Specimen preparation

Variegated leaves of dark green, medium green, light green, and white sectors were fixed with 2% glutaraldehyde in 0.1 M sodium cacodylate buffer, pH 7.4 at four °C overnight, then rinsed with 0.1M sodium cacodylate buffer three times for 15 min. The material was dehydrated through a graded ethanol series for 10 min per step before transferring to n-Butyl glycidyl ether for 1 h. Specimens were infiltrated with Spurr’s resin twice for 1 h, then embedded into resin blocks entirely filled with the resin and polymerised in a hot air oven at 62 °C overnight. To investigate the histology of the leaf punches with an optical microscope, specimen blocks were sectioned into 1 – 1.5 µm thickness using an ultra-microtome (Leica Ultracut UCT-GA-D/E-1/100). The semi-thin sections were stained with 1% Toluidine Blue-O in distilled water and observed under an Olympus CX31 light microscope. To examine chloroplast morphology, specimens were ultrathin-sectioned to 70 – 90 nm with the ultra-microtome and stained with the regular 1% (w/v) uranyl acetate / Lead Citrate staining or Uranyless EM staining (https://www.emsdiasum.com/uranyless), then observed by a Hitachi HT7700 transmission electron microscope.

### Brightfield and Fluorescence microscopy

We obtained 2 × 5 mm patches of variegated leaves comprising the borders of different green shades. We fixed them in three ways: 1% formaldehyde, 2% glutaraldehyde, and 1% formaldehyde plus 2% glutaraldehyde, all in 0.1M sodium cacodylate buffer, pH 7.4, following the procedure in [47, 48]. All materials were degassed with a vacuum pump for 10 min and then kept in a fridge at 4 °C overnight. The next day, we rinsed the specimens with distilled water before embedding them in 3.5% agarose gel, sectioned them into 30 µm with a Leica VT1000S vibratome, and observed the preparations with an Olympus BX51 fluorescence microscope equipped with epifluorescence broadband filters for blue/green (U-MWU2) and green/red (U-MWB2). We used Adobe Photoshop® for contrast and brightness improvement and stitched low-magnification images of cross-sectioned leaves (https://www.adobe.com). Captures at 40 × magnification at different focal planes were stacked with the stacking procedure in the Zerene Stacker app (http://zerenesystems.com). Further scientific artwork was carried out in Affinity Designer v.2 (https://affinity.serif.com).

### Leaf variegation analysis

Images of variegated leaves were captured with a Canon DSLR camera equipped with a 50 mm macro lens and appropriate illumination for shadowless photos. Images were stored at 300 dpi RGB TIFF files. We used the Trainable Weka Segmentation plugin of the Fiji/ImageJ image analysis software (https://imagej.net/software/fiji/, version 2.90/1.54e) that combines machine learning algorithms with a set of selected image features to produce pixel-based segmentations [49, 76]. To this end, we add small samples of the four colour shades (dark green, medium green, light green) and white of the variegated leaf and black background to train the program for defining the five classes. For the training features of the segmentation, we selected only the default Gaussian blur, Hessian, Membrane projections, Sobel filter, and Difference of Gaussians. The resulting pseudo-coloured 8-bit classified image was then converted into an 8-bit RGB colour image. In the next step, we used the colour threshold command and sampled each colour individually to analyse the selected pool of particles thus obtained. Particles of 100 pixels or less were omitted from the measurements. The final results were copied to an MS Excel spreadsheet for further processing and generating charts.

### Photosynthetic pigments

Chlorophyll a (Chl a), chlorophyll b (Chl b) and carotenoid (Caro) content were measured according to [77]. Leaf disc samples of 0.16 cm^2^ were ground and extracted with 10 mL of 80% acetone. The homogenate was filtered using the Whatman® filter paper no.1. The absorbance was analysed by spectrophotometry at 663, 645, and 440 nm. Chlorophyll and carotene contents were computed using the equations as follows:

$$\mathrm{Chlorophyll\ a\ }({\text{g}}/{{\text{m}}}^{2}) = ({12.7{\text{A}}}_{663}- {2.69{\text{A}}}_{645})*{\text{V}}/100*{\text{A}},$$

$$\mathrm{Chlorophyll\ b\ }= \left({22.9{\text{A}}}_{645}\right)- {4.68{\text{A}}}_{663})*{\text{V}}/100*{\text{A}},$$

$$\mathrm{Total\ Chlorophyll }=\mathrm{ Chlorophyll\ a }+\mathrm{ Chlorophyll\ b},$$

$$\mathrm{Carotene }= [{4.69{\text{A}}}_{440}- 0.268({20.20{\text{A}}}_{645}) + {8.02{\text{A}}}_{663}]*{\text{V}}/100*{\text{A}},$$

where V is the volume of the extraction solvent (mL), and A is the area of the leaf sample (cm^2^).

### Mutant mapping and bioinformatics analysis

The genomic regions associated with variegation were identified using the bulked segregant analysis implemented in MutMap version 2.3.3 [51]. TOMAC463 was used as the wild-type and created wild-type like and variegated bulks by pooling genomic DNA from 50 individuals of a reciprocal F_2_ cross. Each reciprocal consisted of 25 individuals. We extracted genomic DNA from these individuals using the CTAB method. DNA samples were sent for whole genome sequencing (WGS) at the China National GeneBank (CNGB) for paired-end sequencing using PCR-free library preparation protocols and sequenced on the MGISEQ-2000 platform. The tomato SL3.0 (NCBI RefSeq assembly GCF_000188115.4) was used as a reference sequence for MutMap. The multiple testing correction implemented in MutMap V.2 was used for Tomato species with default parameters. Only variants with a 99% confidence interval of simulated SNP-index in a region of sliding window analysis line peaked were further investigated to indicate variants linked to causal variegated mutation. The genomics regions linked by a sliding window region were blasted to the tomato (taxid:4081) RefSeq RNA database for identifying genes within the candidate region that exhibited significant sequence similarity to known tomato genes using BLAST + [78]. The polymorphic variations between wildtype (TOMAC463 and F_2_ wildtype bulk) and variegated (M_2_ plant No.443–1 and F_2_ variegated bulk) in candidate regions were identified by using the genome analysis toolkit (GATK) variant discovery pipeline [52] to verify the mutation found by MutMap. The filtering applied for variant quality control following: FS > 60.0, MQ < 40.0, MQRankSum < -12.5, QD < 2.0, QUAL < 30.0, ReadPosRankSum < -8.0, and SOR > 3.0.

The PCR primer was developed to validate the SNP found in candidate *Solanum lycopersicum FtsH*-like protein precursor (LOC100037730), producing the product size of 666 base pairs. The forward primer is ATAATTGCTGGCCCGGAGAA, and the reverse primer is GAGGCAAGCAAGATCATGGA. Two tomato lines with three replicated plants for each line were used to represent normal leaf plants: TOMAC463 (wild-type) and Seedathip3, the parent of TOMAC463. Four lines of the wild-type F_2_ leaf from both reciprocal crosses were used in the validation, which are VTF2 1–4, VTF2 1–35, VTF2 2–11, and VTF2 2–139. Not only F_2_ wild-type leaf, but four lines of F_2_ variegated leaf were also included in the analysis, which are VTF2 1–53, VTF2 1–60, VTF2 2–11, and VTF2 2–139. Five plants of the M_6_ variegated tomatoes generation were also included to represent the inherited variegated trait. Then, the PCR product was sent for Sanger DNA sequencing technique, which was performed on the ABI 3730XL DNA Analyzer at U2bio(Thailand) Co., Ltd. The sequences were aligned and displayed by AliView [79] version 1.28.

The miRNA target binding site investigation involved the utilisation of specialised prediction tools. Specifically, the custom prediction feature of miRDB (https://mirdb.org/custom.html) was employed alongside the target prediction functionality for plant microRNAs provided by TAPIR (https://bioinformatics.psb.ugent.be/webtools/tapir/). To ascertain potential miRNA targets at the SNP position, both the sequence containing substituted bases and the reference sequence were submitted to miRDB and TAPIR for comprehensive analysis. A hundred upstream and a hundred downstream sequences flanking SNP positions were extracted from chromosome 4 of SL3.0. According to a limited model organism for miRNA prediction analysis, all organisms were tested for the potential target in the mRNA target sequence prediction method for tomato sequences with default parameters.

### Statistical analysis

The wild tomato TOMAC463 (*Solanum lycopersicum*) and the M_2_ plant No.443–1(the variegated tomato) were reciprocally crossed to produce reciprocal F_1_ progenies. To obtain the reciprocal F_2_ populations, the F_1_ generations were selfed and grown in a greenhouse in June 2020. A total of 210 seeds of F_2_ generation from each cross were planted in the greenhouse to analyse the segregation ratios of reciprocal crosses in the F_2_ population by using a χ^2^ test of observed values in July 2021. Data management and statistical analyses were performed using MS Excel and IBM SPSS Statistics, v. 26 software. Analysis of variance (ANOVA) and Duncan’s multiple range test were used to compare the means of parameters at *p* = 0.05.

### Supplementary Information

**Supplementary Material 1. **

## Data Availability

The datasets generated and analysed in our study are available upon request.

## Abbreviations

DG: Dark green
InDels: Insertion/deletion polymorphisms
LG: Light green
MG: Medium green
SNP: Single nucleotide polymorphism
WH: White
WT: Wild-type
