# Supplementary material for: Mutation mapping of a variegated EMS tomato reveals an FtsH-like protein precursor potentially causing patches of four phenotype classes in the leaves with distinctive internal morphology
Source: BMC Plant Biol. 2024 Apr 10;24:265. doi: 10.1186/s12870-024-04973-1 (PMC11005157; doi:10.1186/s12870-024-04973-1)
Supplement: Supplementary file 1 — Supplementary Material 1. [file 12870_2024_4973_MOESM1_ESM.docx]

**Supplementary information**

**Suppl. Figure S1.** The phenotype occurs in the variegated plant. a. the variegated pattern displays white-green stripes in sepals, rachides, and petioles; b. the normal petal color; c. the normal fruit color.

**
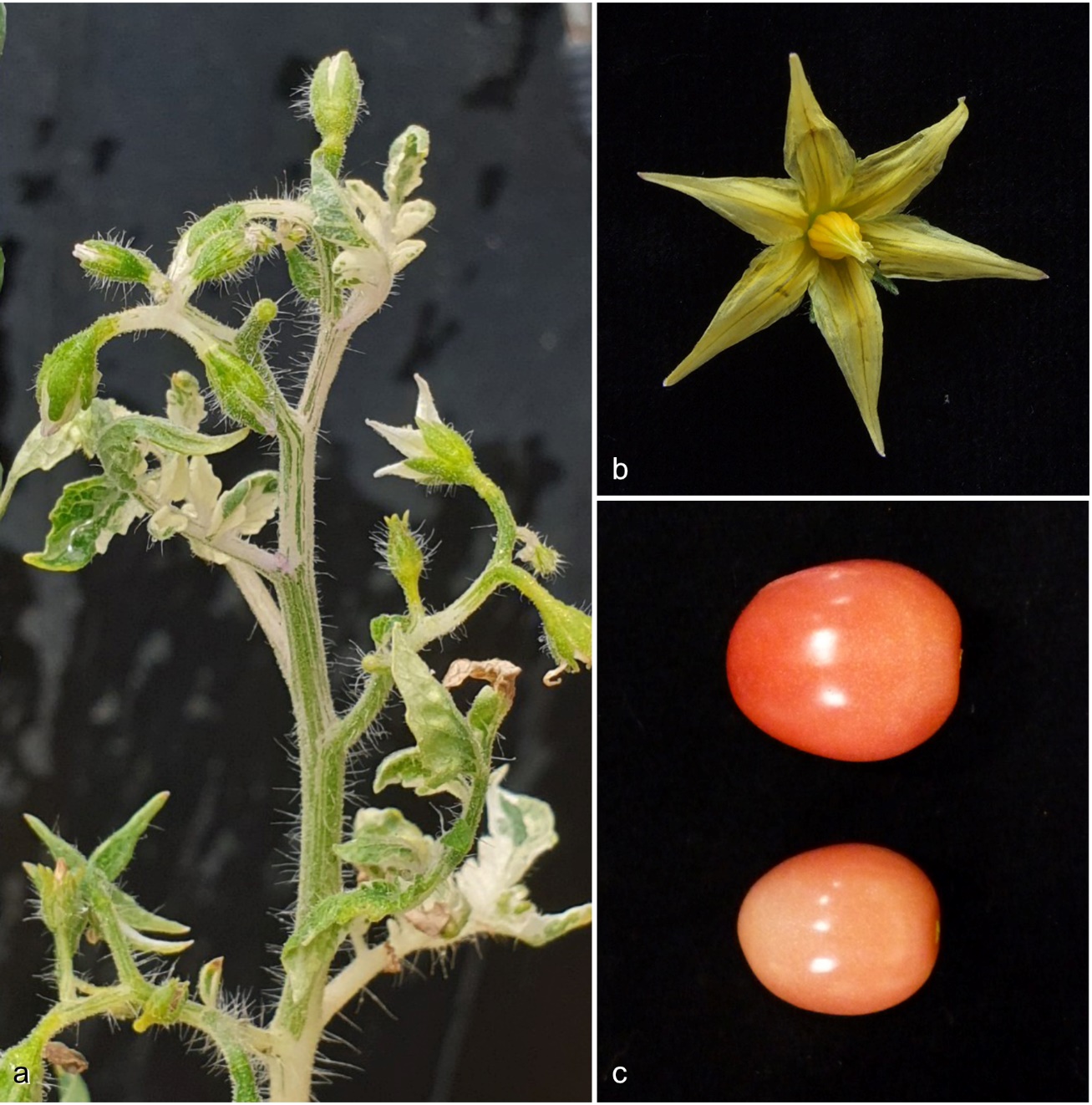
**

**Suppl. Figure S2.** The vibratome sectioning of wild-type (WT) and variegated leaves in fluorescence microscopy. a. cross-section of WT; b. transitional zone between MG and LG sectors which is divided by a midrib; c. the aberrant transition zone from MG toward LG sectors; d. the WH segment toward underneath the palisade cell layer of the DG sector. Arrowhead point at the vein. Arrow indicated the midrib of the leaf. The dashed line divided the extent between MG and LG sectors.

| 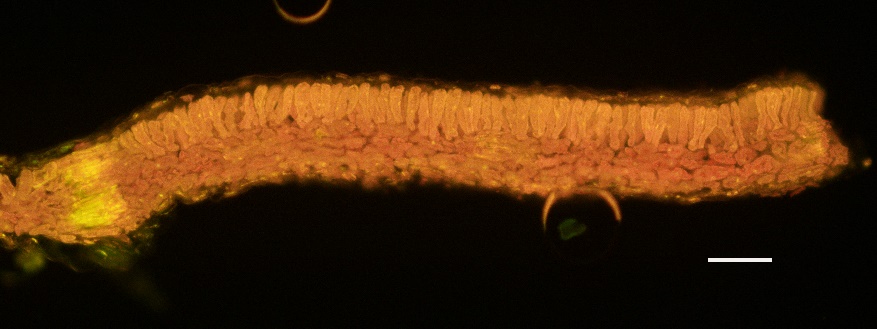  WT  a |
| --- |
| 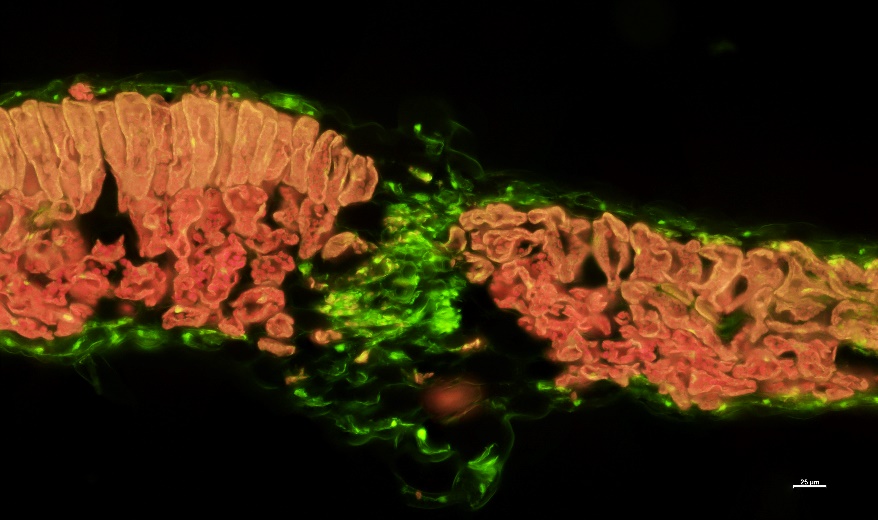  MG  LG  b |
| 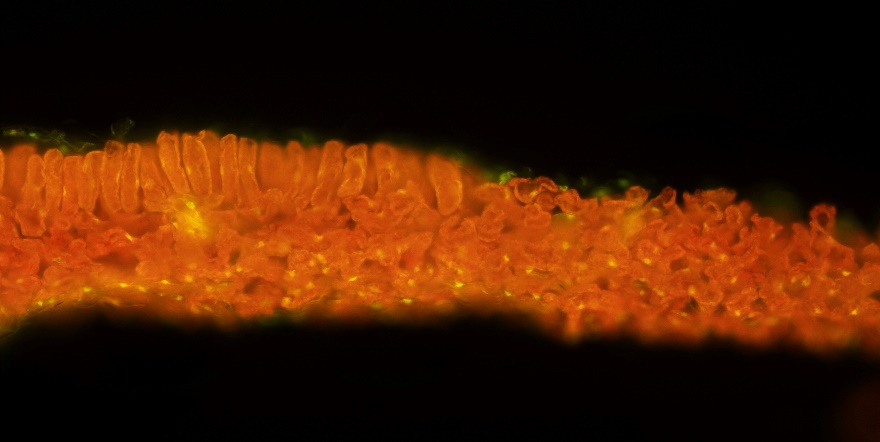  MG  LG  c |
| 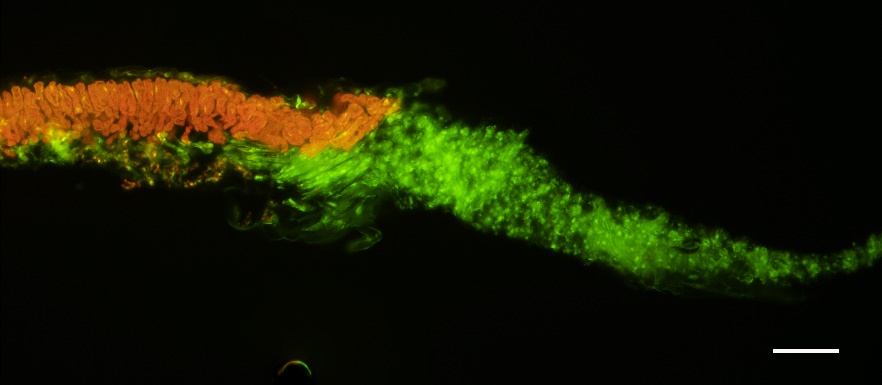  WH  d  DG |

**Suppl. Figure S3.** The sequences alignment result comparing typical and variegated leaf plants at variegated candidate SNP region in *Solanum lycopersicum* FtsH-like protein precursor (LOC100037730) gene.


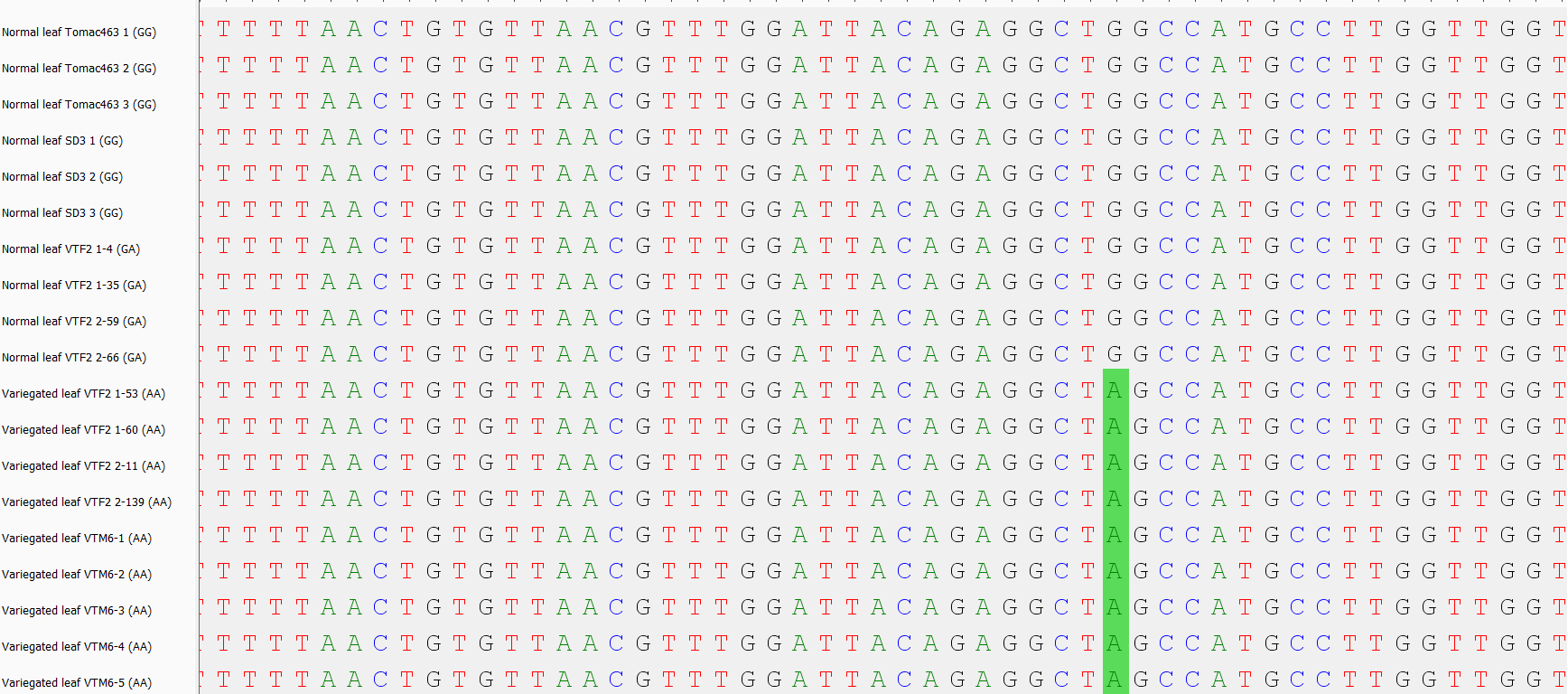


**Suppl. table ST1.** Heatmap of the thickness of leaf layers in the different sectors of the variegated leaves. WT=wild-type, DG=dark green, MG=medium green, LG=light green, WH=white.

| Thickness of leaf layers | | |  |  |  |  |  |
| --- | --- | --- | --- | --- | --- | --- | --- |
|  | leaf thickn | % | pallisade | % | spongy | % | Pall/spongy |
| WT | 158.16 | 100.0 | 54.9 | 100 | 76.5 | 100 | 0.74 |
| DG | 167.13 | 105.7 | 44.9 | 81.8 | 80.1 | 104.7 | 0.57 |
| MG | 118.16 | 74.7 | 37.1 | 67.6 | 59.6 | 77.9 | 0.63 |
| LG | 123.12 | 77.8 | 48.6 | 88.5 | 45.7 | 59.7 | 1.09 |
| WH | 121.82 | 77.0 | 26.8 | 48.8 | 74.1 | 96.9 | 0.39 |
|  |  |  |  |  |  |  |  |
|  |  |  |  |  |  |  |  |
| Number of cells | |  |  |  |  |  |  |
| WT |  |  | 33.8 | 100 | 50.6 | 100 |  |
| DG |  |  | 49.1 | 145.3 | 72 | 142.3 |  |
| MG |  |  | 33.8 | 68.8 | 69 | 95.8 |  |
| LG |  |  | 49 | 145.0 | 68.8 | 99.7 |  |
| WH |  |  | 36.5 | 74.5 | 93.8 | 136.3 |  |

**Suppl. table ST2.**

Overview of particle analysis from leaves following Weka colour segmentation and colour thresholding. Objects smaller than 100 pixels were not considered for the calculations.

|  | class | count | total area | average size | %area | Perimeter | Circularity | Solidity |
| --- | --- | --- | --- | --- | --- | --- | --- | --- |
| Leaflet 1 |  |  |  |  |  |  |  |  |
|  | DG | 15 | 2740 | 182.667 | 0.729 | 106.367 | 0.303 | 0.556 |
|  | MG | 21 | 80199 | 3819 | 21.352 | 400.617 | 0.294 | 0.586 |
|  | LG | 8 | 110172 | 13771.5 | 29.331 | 876.384 | 0.303 | 0.681 |
|  | WH | 31 | 24785 | 799.516 | 6.599 | 195.019 | 0.243 | 0.617 |
|  | sum | 75 |  |  |  |  |  |  |
|  | average | 18.75 |  |  |  |  |  |  |
| Leaflet 2 |  |  |  |  |  |  |  |  |
|  | DG | 16 | 88735 | 5545.938 | 14.977 | 382.987 | 0.46 | 0.756 |
|  | MG | 13 | 201996 | 15538.154 | 34.093 | 581.467 | 0.537 | 0.768 |
|  | LG | 37 | 61361 | 1658.405 | 10.356 | 240.338 | 0.414 | 0.711 |
|  | WH | 10 | 4433 | 443.3 | 0.748 | 154.146 | 0.285 | 0.633 |
|  | sum | 76 |  |  |  |  |  |  |
|  | average | 19 |  |  |  |  |  |  |
| Leaflet 3 |  |  |  |  |  |  |  |  |
|  | DG | 13 | 86552 | 6657.846 | 15.189 | 392.88 | 0.218 | 0.54 |
|  | MG | 5 | 176345 | 35269 | 30.946 | 1127.732 | 0.373 | 0.715 |
|  | LG | 24 | 41845 | 1743.542 | 7.343 | 291.764 | 0.388 | 0.68 |
|  | WH | 11 | 5267 | 478.818 | 0.924 | 178.955 | 0.19 | 0.546 |
|  | sum | 53 |  |  |  |  |  |  |
|  | average | 13.25 |  |  |  |  |  |  |
| Leaflet 4 |  |  |  |  |  |  |  |  |
|  | DG | 21 | 124663 | 5936.333 | 22.082 | 359.502 | 0.455 | 0.746 |
|  | MG | 11 | 154032 | 14002.909 | 27.285 | 498.649 | 0.467 | 0.728 |
|  | LG | 31 | 24539 | 791.581 | 4.347 | 177.187 | 0.436 | 0.728 |
|  | WH | 6 | 3280 | 546.667 | 0.581 | 162.167 | 0.24 | 0.618 |
|  | sum | 69 |  |  |  |  |  |  |
|  | average | 17.25 |  |  |  |  |  |  |
| Leaflet 5 |  |  |  |  |  |  |  |  |
|  | DG | 15 | 73669 | 4911.267 | 22.139 | 413.34 | 0.365 | 0.687 |
|  | MG | 8 | 83848 | 10481 | 25.198 | 622.235 | 0.552 | 0.751 |
|  | LG | 13 | 14866 | 1143.538 | 4.467 | 230.156 | 0.378 | 0.696 |
|  | WH | 10 | 3304 | 330.4 | 0.993 | 127.22 | 0.278 | 0.63 |
|  | sum | 46 |  |  |  |  |  |  |
|  | average | 11.5 |  |  |  |  |  |  |

**Suppl. table ST3.**

| Gene name | Sequence ID |
| --- | --- |
| AtFtsH1 | AT1G50250.1 |
| AtFtsH2 (var2) | AT2G30950.1 |
| AtFtsH3 | NP_850129.1 |
| AtFtsH4 | NP_565616.1 |
| AtFtsH5 (var1) | AT5G42270.1 |
| AtFtsH6 | NP_568311.2 |
| AtFtsH7 | NP_566889.1 |
| AtFtsH8 | NP_1321589.1 |
| AtFtsH9 | NP_568892.1 |
| AtFtsH10 | NP_172231.2 |
| AtFtsH11 | NP_568787.1 |
| AtFtsH12 | NP_565212.1 |
| SlFtsH2 | XP_10323671.1 |
| SlFtsH4 | XP_4245091.1 |
| SlFtsH8 | XP_4246405.1 |
| SlFtsH9 | XP_4240393.1 |
| SlFtsH10 | XP_10312354.1 |
| SlFtsH11 | XP_4234177.1 |
| SlFtsH12 | XP_4232810.1 |
| SlFtsH protease | NP_1234191.1 |
| SlFtsH-like protein precursor | NP_1234196.2 |

The list of 9 tomato and 12 Arabidopsis *FtsH* genes used in phylogenetic study.
